# Integration of Multiomics Data Reveals Selection Characteristics of *ITGB1* That Are Associated with Size Differentiation in Pigs

**DOI:** 10.3390/ijms26041569

**Published:** 2025-02-13

**Authors:** Guandong Wu, Miao Yu, Tianxin Liu, Dongjie Zhang, Yang Chang, Zhonghua Liu, Di Liu, Chunzhu Xu

**Affiliations:** 1 Key Laboratory of Animal Cellular and Genetics Engineering of Heilongjiang Province, Engineering Research Center of Intelligent Breeding and Farming of Pig in Northern Cold Region, College of Life Science, Northeast Agricultural University, Harbin 150030, China; s230902039@neau.edu.cn (G.W.); s230901001@neau.edu.cn (M.Y.); s220901030@neau.edu.cn (T.L.); changy051@neau.edu.cn (Y.C.); liuzhonghua@neau.edu.cn (Z.L.); 2 Institute of Animal Husbandry, Heilongjiang Academy of Agricultural Sciences, Harbin 150086, China; djzhang8109@163.com

**Keywords:** Min pig, selection characteristics, transcriptome, resequencing, *ITGB1*

## Abstract

Min pigs, a prominent local breed from Northeast China, have diverged into two distinct breeds, Ermin (EM) pigs and Hebao (HB) pigs, through prolonged natural and artificial selection. Although these two breeds exhibit distinct differences in body size, the genetic mechanisms underlying this variation remain poorly understood. In this study, we performed whole-genome resequencing and transcriptome analysis on EM and HB pigs to elucidate the genetic basis of body size variation in Min pigs through genome-wide selection signal analysis and the identification of differentially expressed genes (DEGs). The analysis of genetic diversity and population genetic structure across 14 pig breeds revealed that, compared with other breeds, Min pigs present relatively high genetic diversity and a unique genetic structure. Notably, EM pigs exhibited significant genetic differentiation from HB pigs. Integrated analysis of whole-genome resequencing and transcriptome data revealed candidate genes associated with body size variation in Min pigs, including *ENPP1*, *ENPP3*, *SPP1*, *CLU*, *ITGA11*, *ITGB1*, *IQGAP2*, *BMP7*, and *F2RL2*. These genes are enriched primarily in pathways related to ECM–receptor interactions; pantothenate and CoA biosynthesis; starch and sucrose metabolism; nicotinate and nicotinamide metabolism; pyrimidine metabolism; nucleotide metabolism; cellular responses to lipids; biomineral tissue development; biomineralization; and other pathways related to cell signaling, metabolic responses, lipid deposition, and skeletal development. Notably, *ITGB1* on chromosome 10 showed strong positive selection in EM pigs, with an SNP locus exhibiting a significant G/A allele frequency difference between EM pigs (G = 52.94%, A = 47.06%) and HB pigs (G = 0%, A = 100%). Our findings suggest that Min pigs potentially modulate lipid metabolism efficiency in adipose tissue through variations in the expression of the *ITGB1* gene, potentially contributing to body size differences. These results provide new insights into the genetic mechanisms underlying body size variation in domestic pigs and serve as a valuable reference for identifying and breeding pig breeds with distinct body sizes.

## 1. Introduction

Domestic pigs are a major source of meat production worldwide [1]. Prolonged artificial selection and breeding programs have resulted in extensive phenotypic diversity in pig body size, making pigs ideal model organisms for studying the molecular mechanisms underlying body size variation, as well as insights into human height and obesity [2]. Min pigs, a local breed from Northeast China, are divided into two body size types: medium-to-large EM pigs and small HB pigs [3]. Historical records suggest that both EM and HB pigs originated from North China black pigs and that they share a common genetic background. However, they exhibit more than a twofold difference in body weight, leading to pronounced differences in body size. The average body weight of EM pigs is 227.10 ± 8.70 kg, with an average body length of 152.20 ± 0.81 cm. In contrast, HB pigs have an average body weight of 93.30 ± 0.76 kg and an average body length of 124.50 ± 0.47 cm [4]. In recent years, the molecular mechanisms regulating body size or weight in domestic pigs have been widely studied, and numerous key genes associated with body size variation in domestic pigs have been identified. For example, Fan et al. identified the *VRTN* gene, which influences body size by modulating the number of vertebrae in pigs [5]. Yang et al. identified 32 candidate genes related to body size variation among different pig breeds, including *ADRB3*, *IGFR1*, *ITGA2*, *ITGA8*, *CAMK2D*, and *ROCK2* [6]. Reyer et al. validated associations between candidate genes related to obesity traits in pigs, such as *SLC27A6*, *SPARC*, *BBS7*, and *MC4R*, which were significantly associated with fat deposition, the lean meat ratio, and the growth rate [7]. Nevertheless, the molecular mechanisms responsible for body size variation in Min pigs remain poorly understood.

Advances in high-throughput technologies and reduced costs have made high-throughput sequencing one of the fastest and most effective methods for detecting genetic variation and identifying functional genes in molecular breeding [8,9]. Currently, methodologies, including whole-genome selection signaling and transcriptome sequencing, are widely used to assess genetic diversity and population differentiation among a variety of animals. These methods have facilitated significant progress in uncovering the mechanisms underlying economically important traits in domesticated animals, such as sheep [10], cattle [11], chickens [12,13,14,15], fish [16,17,18,19], and pigs [20,21,22,23,24]. For example, Huang et al. demonstrated that genetic introgression from Chinese indigenous pig breeds enhanced fertility and immunity in European commercial pigs [25], and Zhao et al. identified six key genes associated with cold adaptation in chickens [26]. However, integrative multi-omics studies identifying key genes underlying body size variation and genetic differentiation in Min pigs have not yet been reported. In this study, we identified key candidate genes and metabolic pathways associated with body size variation in Min pigs through an integrated analysis of whole-genome resequencing and transcriptome data. We analyzed EM pigs and HB pigs, which share a common genetic origin but exhibit significant differences in body size. Our comprehensive approach examined the selection patterns, tissue-specific differential expression, and population genetic characteristics of these candidate genes to elucidate the genetic mechanisms underlying body size variation in Min pigs. These findings provide valuable insights into the genetic evolution of body size traits and offer a scientific foundation for targeted breeding strategies to develop pig breeds optimized for meat production or medicinal use. Furthermore, our study establishes a theoretical basis for advancing the understanding of the molecular mechanisms involved in pig growth, development, and metabolism.

## 2. Results

### 2.1. Population Genetic Diversity Analysis of 14 Pig Breeds

The whole-genome resequencing of 341 individuals from 14 pig breeds (large white (LW), Landrace (LR), Duroc (DRC), wild boar (WB), Tongcheng (TC), Shaziling (SZL), Bamaxiang (BMX), Rongchang (RC), Erhualian (EHL), Dongshan (DS), Ganxi (GX), and Jinhua (JH), as well as HB and EM) produced high-quality genotyping data for 24,079 SNP loci. Genetic diversity indices were calculated for each pig population on the basis of genome-wide SNP data. The results revealed that the nucleotide diversity (π), expected heterozygosity (He), observed heterozygosity (Ho), polymorphism information content (PIC), and minor allele frequency (MAF) values of the HB and EM pig populations were greater than those of other Asian local pig breeds and Asian wild boars but slightly lower than those of the three commercial pig populations. The effective population size (Ne), the proportion of polymorphic markers (PN), and $r^{2}$ values for the HB and EM pig populations were similar to those of the commercial pig populations (Table 1). These findings suggest that the Min pig population has experienced relatively intense selection pressures, but less stringent breeding management has led to lower genetic diversity than that found in commercial pig breeds.

### 2.2. Genetic Structure Analysis of EM and HB Pigs

**Figure 1:**
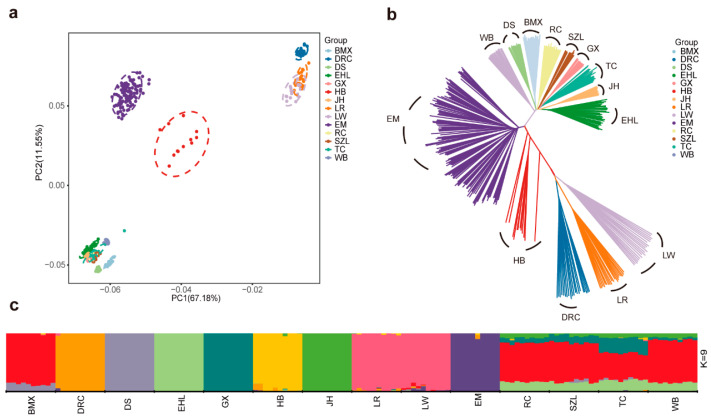
Population genetic structure characteristics of 14 pig breeds. (**a**) PCA of 341 pigs analyzed in this study. PC1 and PC2 represent the first and second principal components, respectively. (**b**) Phylogenetic tree constructed via the NJ method, where each color denotes a different breed. (**c**) Population genetic structure inferred by ADMIXTURE, assuming nine ancestral clusters (K = 9). Each color represents an ancestral group, with each vertical line corresponding to an individual pig. Breed abbreviations are provided in Table 1.

PCA on 341 pigs from 14 breeds revealed that the first principal component (PC1) explained 67.18% of the total variance and the second principal component (PC2) explained 11.55%, effectively separating the Asian and commercial pig populations, both individually and collectively. The analysis further revealed a distinct genetic separation between the EM and HB pig populations (Figure 1a). Phylogenetic analysis revealed that individuals from the same breed clustered together, with clear genetic separation between commercial and Asian breeds. The EM and HB pigs were positioned intermediately between these groups (Figure 1b), which is consistent with the PCA results (Figure 1a,b). ADMIXTURE analysis of 140 individuals from 14 breeds (10 randomly selected pigs per breed) identified 9 as the optimal number of ancestral populations (K), minimizing the cross-validation error to 0.48535. The EM and HB pigs formed distinct population clusters (Figure 1c). Overall, these results indicate that the EM and HB pig populations have unique genetic structures and show significant genetic differentiation from each other.

### 2.3. Selective Sweeps for Body Size Variation Adaptation

**Figure 2:**
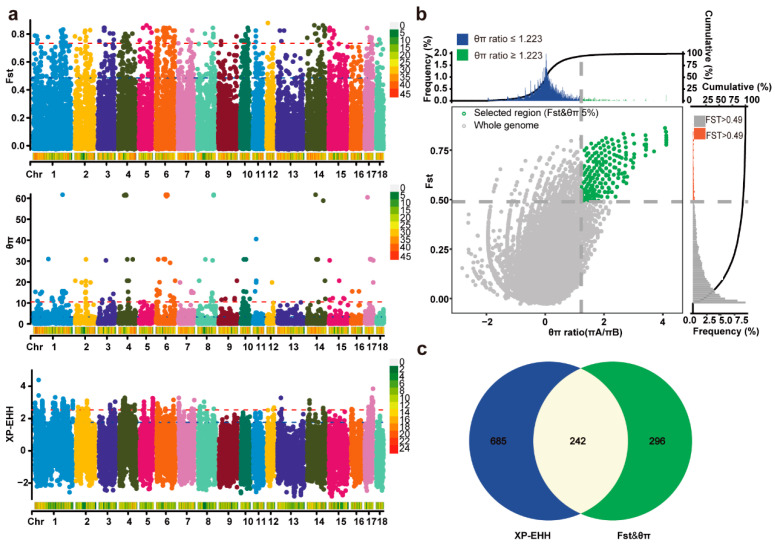
Genome-wide selective sweep signals identified in EM pigs compared with those in HB pigs. (**a**) Manhattan plot illustrating genome-wide selective signatures based on F_ST_, log_10_ (θπ ratio), and XP-EHH between EM and HB pigs. The analysis was conducted using 500 kb windows with a 50 kb step size. The horizontal red line indicates the top 1% cutoff, while the horizontal blue line represents the top 5% cutoff. (**b**) Final selection regions identified via the F_ST_ and θ_π_ statistical methods. Points to the right of the vertical dashed line (corresponding to the top 5% of the empirical log_2_ (*π_A_*/*π_B_*) ratio distribution, with a threshold of 1.223) and above the horizontal dashed line (representing the top 5% of the empirical F_ST_ distribution, with a threshold of 0.49) were identified as selected regions for the EM pig population (green dots). (**c**) Venn diagram showing the overlap of genes identified by both the F_ST_ & log_10_ (θ_π_ ratio) and XP-EHH methods, with 242 genes shared between the two methods highlighted.

To investigate potential genes influencing body size variation in Min pigs, we employed three genome-wide selection signal analysis methods, namely, nucleotide diversity ratio (θ_π_), F-statistics (F_ST_), and Cross-population Extended Haplotype Homozygosity (XP-EHH), to identify differentially selected regions between the EM and HB pig populations. The results indicated that 538 genes were annotated in the region where the joint analysis of F_ST_ and θ_π_ exceeded the top 5% threshold (Figure 2b) (Table S2). The XP-EHH method identified 21,799 SNP loci, with 927 genes annotated in the region meeting the top 5% positive threshold (Figure 2a) (Table S3). In total, 242 genes were identified via all three selection signal analysis methods (Figure 2c).

### 2.4. Identification of Differentially Expressed Genes Between EM and HB Pigs

**Figure 3:**
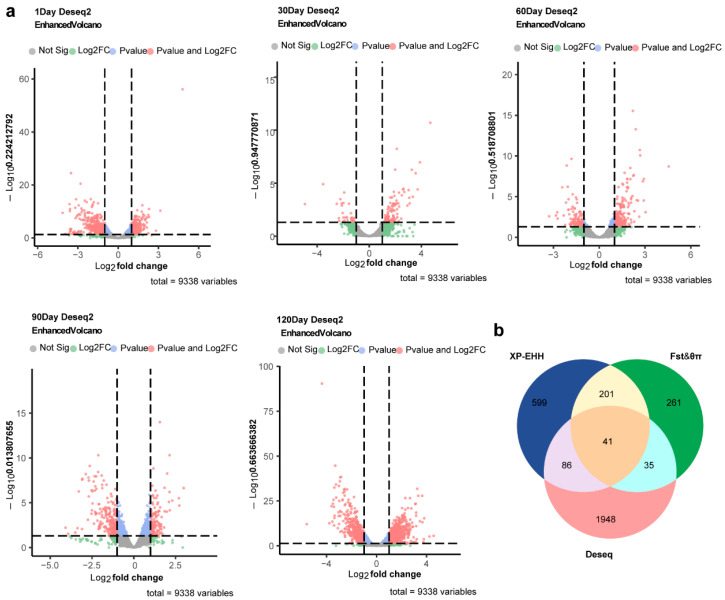
Volcano map and selective signal analysis of DEGs and Venn map of DEGs between EM pigs and HB pigs at the same stage. (**a**) Each volcano plot displays gene expression changes at different time points (1 day, 30 days, 60 days, 90 days, and 120 days). The *x*-axis represents the log2 fold change, and the *y*-axis shows the −Log10 *p*-value. Genes are color-coded as follows: gray (nonsignificant), blue (significant log2 fold change only), green (significant *p*-value only), and red (both significant). The vertical dashed lines indicate a 2-fold change threshold, and the horizontal dashed line represents the significance threshold at *p* < 0.05. The total number of variables analyzed is 9338. (**b**) Venn diagram displaying the overlap of genes identified by XP-EHH, the F_ST_/θ_π_ ratio, and DESeq2 differential expression analysis. A total of 41 genes were identified by all three methods, as shown in the central overlapping region.

Differential gene expression analysis between EM and HB pigs at ages 1, 30, 60, 90, and 120 days identified 652, 120, 311, 367, and 1438 differentially expressed genes, respectively (Tables S4–S8). The cumulative analysis across all the age groups revealed 2110 DEGs associated with growth and development from 1 to 120 days (Figure 3a). Furthermore, 41 genes potentially contributing to body size variation in Min pigs were identified through a combination of genome-wide selection signals and differential gene expression analyses (Figure 3b).

### 2.5. Screening for Candidate Genes Associated with Body Size Variation

**Figure 4:**
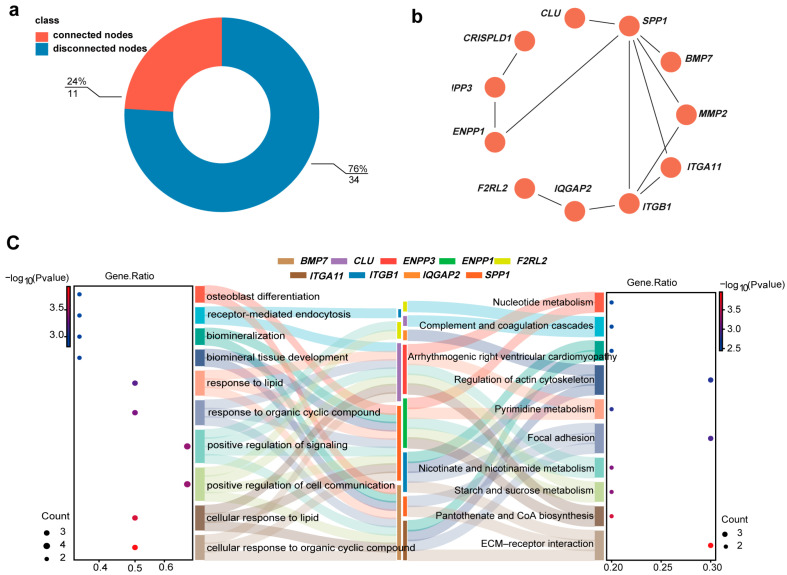
Candidate gene interaction network and enrichment analysis of KEGG and GO pathways. (**a**) Donut chart showing the proportion of genes within the PPI network. Red indicates 11 genes that are connected (24% of the total), whereas blue represents 34 disconnected genes (76% of the total). (**b**) PPI network of the 11 connected genes. Each node represents a gene, with colored circles denoting individual genes and black lines indicating direct interactions between them. (**c**) Enrichment analysis of the top 10 pathways from KEGG and GO analysis for the 11 selected genes. The left panel shows enriched GO terms, and the right panel displays KEGG pathways. The colors in the middle Sankey chart represent the specific genes involved in each pathway.

Protein network interaction analyses of 41 candidate genes implicated in Min pig body size variation revealed that 11 key genes, *ENPP1*, *ENPP3*, *SPP1, CLU*, *ITGA11*, *ITGB1*, *IQGAP2*, *BMP7*, *F2RL2*, *MMP2*, and *CRISPLD1,* significantly influenced this trait (Figure 4a,b). KEGG and GO analyses of these genes revealed 327 significantly enriched GO terms (*p* < 0.05) (Table S9), with the top 10 enriched terms related to lipid deposition, mineral tissue development, and cellular communication. KEGG enrichment analysis revealed 19 significantly enriched pathways (*p* < 0.05) (Table S10), including ECM–receptor interaction, pantothenic acid and coenzyme A biosynthesis, starch and sucrose metabolism, nicotinic acid and nicotinamide metabolism, focal adhesion, pyrimidine metabolism, the regulation of the actin cytoskeleton, arrhythmogenic right ventricular cardiomyopathy, complement and clotting cascades, and nucleotide metabolic pathways. Nine of these genes, *ENPP1*, *ENPP3*, *SPP1*, *CLU*, *ITGA11*, *ITGB1*, *IQGAP2*, *BMP7*, and *F2RL2,* were found within these pathways (Figure 4c).

### 2.6. Selection Patterns of Candidate Genes Associated with Body Size in Domestic Pigs

To further elucidate the selection patterns of the nine genes *ENPP1*, *ENPP3*, *SPP1*, *CLU*, *ITGA11*, *ITGB1*, *IQGAP2*, *BMP7*, and *F2RL2* identified in Min pigs, we selected four pig breeds with distinct body size differences, namely, EM, WZS, TIB, and DRC, for selection pressure analysis. The results indicated that the *ITGB1* gene (ω = 3.49525) and *CLU* gene (ω = 1.15395) experienced varying levels of positive selection, with several selected SNP loci identified. In contrast, the remaining seven genes underwent varying degrees of negative selection (ω < 1) (Table 2).

### 2.7. Quantitative Real-Time PCR (qRT–PCR) Validation in Different Tissues and Organs of Min Pigs

**Figure 5:**
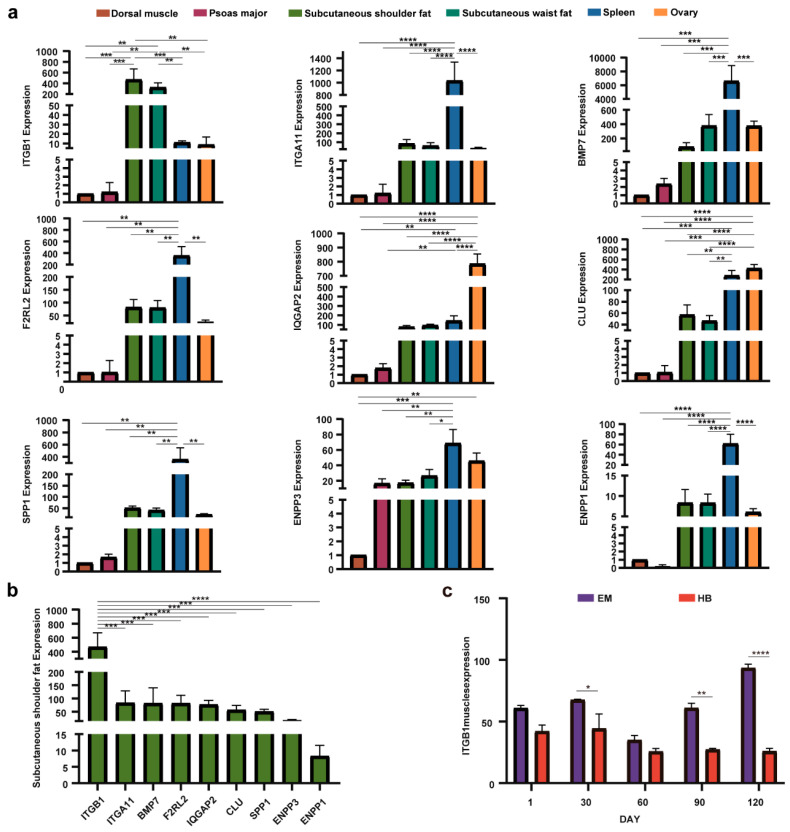
qRT–PCR results for candidate genes in different tissues in EM pigs and *ITGB1* timing analysis results for EM and HB pigs. (**a**) Relative expression levels of selected genes across various tissues in EM pigs. The *y*-axis represents gene expression levels relative to those in the dorsal muscle tissue, and the *x*-axis represents six different tissues: the dorsal muscle, psoas major, subcutaneous shoulder fat, subcutaneous waist fat, spleen, and ovary. (**b**) Expression levels of selected genes in the subcutaneous shoulder fat tissue of EM pigs. The *y*-axis represents gene expression levels in this tissue, whereas the *x*-axis shows the names of the different genes. (**c**) Expression of the *ITGB1* gene at different growth stages in EM and HB pigs. The *x*-axis represents the age in days (1, 30, 60, 90, and 120) for EM and HB pigs, and the *y*-axis represents the expression levels of *ITGB1* (* *p* ≤ 0.05, ** *p* ≤ 0.01, *** *p* ≤ 0.001, **** *p* ≤ 0.0001).

To further elucidate the expression profiles of the nine candidate genes in various tissues of EM pigs, qRT–PCR was used to assess the relative expression levels of these genes across six tissues: the dorsal muscle, psoas major, subcutaneous shoulder fat, subcutaneous waist fat, ovary, and spleen. The results revealed that the *ITGB1* gene presented the highest expression in subcutaneous shoulder fat, whereas *IQGAP2* and *CLU* presented peak expression in the ovary. Additionally, *ITGA11*, *BMP7*, *F2RL2*, *SPP1*, *ENPP3*, and *ENPP1* presented the highest expression levels in the spleen. Notably, *ITGB1* expression was significantly greater in subcutaneous waist fat than in subcutaneous shoulder fat and in other tissues (Figure 5a,b). Furthermore, throughout the growth and development period from 1 to 120 days, *ITGB1* expression in EM pigs remained consistently higher than that in HB pigs, with a tendency toward increased expression in EM pigs as they aged (Figure 5c).

### 2.8. Haplotype Analysis of ITGB1 EM and HB Pigs

**Figure 6:**
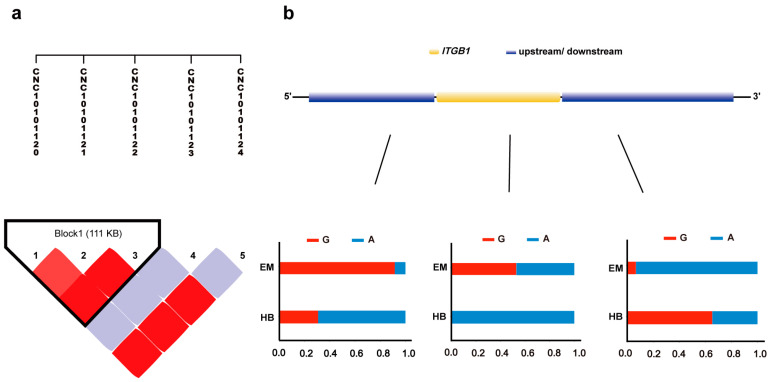
*ITGB1* haplotype analysis. (**a**) Haplotype analysis of SNPs in the upstream and downstream regions of the *ITGB1* gene in EM pigs. LD between SNPs is represented by squares, with red indicating higher LD values and white indicating lower LD values. Each square shows the LD value between a pair of SNPs, with thin lines linking the SNPs. (**b**) Structure of the *ITGB1* gene and the allele frequencies of three SNPs in the *ITGB1* gene and its flanking regions in the EM and HB pig populations. The yellow region represents the *ITGB1* gene, and the blue regions indicate the upstream and downstream sequences. Bar charts show the allele frequencies of each SNP (G and A alleles) in the EM and HB pigs.

Haplotype analysis and allele frequency calculations for SNP loci in the upstream and downstream regions of the *ITGB1* gene were conducted in EM and HB pigs to explore the associations between *ITGB1* gene variation and body size differences between these two Min pig populations. An LD block of the *ITGB1* gene, located on chromosome 10 in EM pigs, was constructed, revealing that the LD block in EM pigs contained three SNP loci (Figure 6a). Notably, the SNP locus CNC10101121 exhibited a G/A polymorphism in the EM pig population, with allele frequencies of G = 52.94% and A = 47.06%, whereas in the HB pig population, the allele frequencies were G = 0% and A = 100% (Figure 6b).

## 3. Discussion

### 3.1. The Unique Genetic Structure of Min Pigs

In this study, we analyzed the population genetic diversity and genetic structure of 14 pig breeds, including EM and HB pigs, via whole-genome resequencing data. Our findings reveal that Min pigs exhibit relatively high genetic diversity and possess a unique genetic structure compared with other pig breeds. Additionally, the EM and HB pig populations were genetically intermediate between commercial pigs and Asian local pig breeds. These results were consistent with the findings of Liu et al., who confirmed the unique genetic structure of Min pigs via the use of paternal and maternal genetic markers [27]. Comprehensive analysis suggested that genetic introgression from European pig breeds significantly increased the genetic diversity of Min pigs, whereas artificial selection and adaptive evolution to environmental conditions are key factors in shaping the unique genetic structure of Min pig populations [28,29].

Furthermore, this study revealed substantial genetic differentiation between the EM and HB pig populations, identifying significant selection pressures and tissue-specific expression differences in the *ITGB1* gene between the two Min pig populations. These findings are consistent with our previous research, which demonstrated that the PLIN1 protein regulates small body size in HB pigs through a distinct selection pattern during artificial directional breeding [4]. During the Qing Dynasty, with the northward migration of Han people (including intermarriage between Han and Manchu populations), medium- and large-sized domestic pigs from northern China were introduced to northeastern China. Over 300 years of natural and artificial selection led to the development of the current EM and HB pig breeds [30]. The Manchu ancestors of the Qing Dynasty, inheritors of Hongshan culture, preferred to use whole pigs in ancestral rituals, with the selection of mature small pigs considered more devout [31]. Therefore, Manchu sacrificial practices likely served as key cultural drivers for the selective breeding of HB pigs to achieve smaller body sizes [4]. According to the research above, analysis suggests that body size variation in Min pigs may be closely linked to their origins and regional sacrificial culture.

### 3.2. Biological Process of Body Type Variation in Domestic Pigs

Growth and development are pivotal economic traits in pig breeding, and their regulation involves complex interactions among multiple genes. Abnormal skeletal development can lead to changes in body size, as observed in purebred dogs, where selection for the *IGF1* and *COL11A2* genes is associated with differences in height [32]. In this study, the identified candidate genes were found to play roles in skeletal development. For example, *ENPP1* and *ENPP3* are involved in starch and sucrose metabolism and affect bone mineralization and soft tissue calcification by regulating pyrophosphate levels [33]. The *SPP1* and *BMP7* genes are critical in lipid metabolism and skeletal development. As a member of the TGF-β superfamily, BMP7 promotes morphogenesis and cell differentiation, including the development of adipose tissue [34]. The SPP1 protein is involved in osteoclast function and contributes to skeletal muscle development in cattle and ducks [35,36]. ITGB1 influences osteoblast function, including cell compaction and mineralized bone nodule formation by participating in fibronectin fibrillogenesis and cell-mediated matrix assembly in muscle satellite cells [37]. Notably, the *ITGB1* gene was identified as being under strong positive selection in this study, and selection pressure analyses offer valuable insights into the gene’s evolutionary trajectory [38], highlighting its importance in the genetic evolution of Min pigs.

The *ITGB1* gene encodes the integrin β-1 subunit, a key component of the integrin family, which forms heterodimers with various ligands and participates in intracellular signaling to regulate diverse cellular processes [39]. Notably, *ITGB1* plays a pivotal role in promoting the differentiation of adipose-derived mesenchymal stem cells into cartilage [40,41]. In our study, *ITGB1* expression was significantly upregulated in EM pigs during growth and development, particularly in adipose tissue, suggesting its crucial role in adipose tissue development and function. Furthermore, integrin–extracellular matrix interactions have been recognized as key regulators of adipose tissue function and systemic metabolism [42]. In the haplotype analysis conducted in this study, an SNP in *ITGB1* had a G allele frequency of 52.94% and an A allele frequency of 47.06% in EM pigs, whereas the G allele was absent (0%), and the A allele was fixed (100%) in HB pigs. This substantial difference in allele frequency could significantly influence ITGB1 expression or protein function, potentially playing a crucial role in the genetic evolution of Min pigs.

In summary, *ITGB1* is a significant candidate gene potentially associated with body size variation in Min pigs through its regulation of lipid deposition and bone development. These findings provide potential genetic targets for breeding pigs with varying body sizes, which could guide breeding practices to improve the growth performance and meat quality of domestic pigs. Future functional studies on *ITGB1* at both the adipocyte and organismal levels in Min pigs are planned to elucidate the molecular mechanisms by which it regulates lipid metabolism in porcine adipocytes. These findings could provide new molecular markers for the precise genetic improvement of domestic pigs.

## 4. Materials and Methods

### 4.1. Sample Preparation

From 2018 to 2022, muscle tissue samples were collected for whole-genome resequencing from 119 EM pigs at a national conservation farm in Lanxi County, Suihua city, Heilongjiang Province (126°13′17.312″ E, 46°21′48.169″ N), and from 13 HB pigs at a conservation farm in Lingyuan city, Liaoning Province (119°40.969″ E, 41°17′58.808″ N). Five sampling time points were established at each farm: 1 day (newborn), 30 days, 60 days, 90 days, and 120 days. At each time point, three muscle tissue samples were collected from each farm, totaling 15 EM and 15 HB samples for transcriptome sequencing. For adult EM and HB pigs, additional samples were collected from six tissues, including the dorsal muscle, psoas major, subcutaneous shoulder fat, subcutaneous waist fat, ovary, and spleen, yielding 18 samples per group. Immediately after collection, the samples were flash-frozen in liquid nitrogen and stored at −80 °C upon arrival at the laboratory.

### 4.2. Genomic DNA Extraction, Genotyping, and Quality Control

Genomic DNA was extracted via the phenol–chloroform method, and DNA quality was assessed via UV spectrophotometry with a NanoDrop 2000 instrument (Thermo Scientific, Shanghai, China) and agarose gel electrophoresis (DYY-6C & DYCP-31BN equipment; Beijing Liuyi, Beijing, China). DNA samples with OD values between 1.8 and 2.0 were diluted to a final concentration of 20 ng/μL. A total of 132 genomic DNA samples from EM and HB pigs were genotyped via the KPS Porcine Breeding Chip v1 50K (Compass Biotechnology Co., Ltd., Beijing, China). The qualified DNA samples were genotyped using KPS Porcine Breeding Chip v1 50K, which is a chip designed based on the application of genome selection breeding. The chip meets the requirements of genome selection breeding for the chip that “involving as many traits as possible, distribute evenly on the genome, which has high polymorphism and strong specificity” [29]. In the chip design, the single-nucleotide polymorphism (SNP) variation loci related to the growth, reproduction, and meat quality traits of Chinese local pig breeds were obtained by resequencing technology. The credibility of SNP loci was scored according to the requirements of MAF and genome average distribution. The missing genotypes were imputed via Beagle (v5.20) software [43,44]. Finally, the chip was customized by Illumina optical fiber microbead technology. To reveal the fine population structure of Min pigs from a global perspective, we extracted the common sites from the KPS porcine breeding chip v1 (including EM, HB) and Illumina 60K SNPs chip, including LW, LR, DRC, WB, TC, SZL, BMX, RC, EHL, DS, GX, and JH, according to the chromosome information and physical location information of SNP loci, which were related to common SNP sites in pigs. The SNP positions within a chromosome were based on the current pig genome assembly (*Sus scrofa* 10.2). A total of 24,279 sites were extracted for follow-up analysis. Data from these breeds were integrated at common loci [45,46] and merged with the KPS Porcine Breeding Chip v1 50K data for comprehensive analysis. The quality control (QC) of the combined SNP data was conducted via PLINK (v1.90) software [47] with the following criteria: SNPs located on autosomes were retained; individuals with a call rate below 0.90 were excluded; SNPs with a call rate less than 0.90 were removed; SNPs with an MAF below 0.01 were eliminated; and SNPs not in Hardy–Weinberg equilibrium with a *p* value less than 1 × 10^−6^ were removed.

### 4.3. Analysis of Genetic Diversity in 14 Pig Breeds

After the QC of the SNP microarray data, we analyzed the genetic diversity of the conserved Min pig population via PLINK (v1.90). This analysis included calculations of π, He, Ho, PIC, MAF, PN, and linkage disequilibrium (LD) values. We also estimated the Ne on the basis of linkage disequilibrium levels [48].

### 4.4. Population Genetic Structure Analyses

On the basis of the integrated genome-wide SNP data, principal component analysis (PCA) was conducted on 341 samples from 14 Eurasian pig breeds (LW, LR, DRC, WB, TC, SZL, BMX, RC, EHL, DS, GX, JH, EM, and HB) via PLINK (v1.90). The first two principal components (PC1 and PC2) were visualized to distinguish the population genetic structure via the R packages “barlot” (v4.3.2) and “ggplot2” (v3.3.5). Population genetic structure analysis was further performed on a subset of 140 samples (with 10 female individuals randomly selected per breed) from the 14 Eurasian pig breeds via ADMIXTURE (v1.30) [49], with visualization completed in TBTOOLS (v2.119) [50]. A neighbor-joining (NJ) phylogenetic tree was constructed with TASSEL (v5) [51] and visualized in iTOL (v5) [52].

### 4.5. Identification of Selective Sweeps Associated with Body Size Variation

To explore the genetic distinctions between the EM and HB pig populations, we conducted interpopulation selection signal analyses via both the combined F_ST_ and θ_π_ ratio joint statistical approach and the XP-EHH method. These analyses were applied to the merged and QC genome-wide SNP data. Pairwise F_ST_ and θ_π_ calculations were performed with VCFTOOLS (v1.13) [53] using a sliding window of 500 kb and a step size of 50 kb. The overlapping regions, corresponding to the top 5% of SNPs identified by both methods, were designated the final selection regions. The joint F_ST_ and θ_π_ selection signal regions were visualized via an R package. For the XP-EHH detection method, genotype phasing was conducted with BEAGLE (v5.0) software, followed by the calculation of XP-EHH scores for each autosomal SNP via SELSCAN (v1.30) [54]. The top 5% of XP-EHH scores were used as the threshold for significant selection signals in the forward direction.

### 4.6. Screening of Differentially Expressed Genes Between EM and HB Pigs

The FPKM values obtained from transcriptome sequencing were used to represent the expression levels of the corresponding unigenes. Gene expression data from two Min pig breeds at five different growth stages were analyzed to identify DEGs between pig breeds of the same age. The DESeq2 tool (v1.46) on the Meiji BioCloud platform (https://cloud.majorbio.com/, accessed on 25 April 2024) [55] was employed for this analysis, applying screening criteria of |log2Fold Change| > 1 and a Q value < 0.05 to effectively control the false-positive rate. To identify differentially expressed candidate genes within the selection signal scanning region, we used the STRING platform (https://cn.string-db.org/, accessed on 28 April 2024) [56,57] to construct a protein–protein interaction (PPI) network and performed network analysis on selected DEGs via the MCODE tool in Cytoscape (v3.9.1) [58].

### 4.7. Functional Enrichment of Candidate Genes

Functional and pathway enrichment analyses of candidate genes were performed via the KOBAS (v3.0) database (http://bioinfo.org/kobas/genelist/, accessed on 2 May 2024) [59] for Gene Ontology (GO) terms and Kyoto Encyclopedia of Genes and Genomes (KEGG) pathways. GO terms and KEGG pathways with *p* values < 0.05 were considered significant. The visualization of the results was carried out via the R packages “clusterProfiler” (v 4.12.6), “org.Hs.eg.db” (v4.3.0), “enrichplot” (v1.9.3), and “ggplot2”.

### 4.8. Selection Pattern of Body Size Candidate Genes in Four Pigs

To elucidate the selection patterns of the nine candidate genes identified in Min pigs, we performed a selection pressure analysis for each gene via EasyCodeML (v1.4) [60]. Genomic and annotation data for three additional pig breeds—Wuzhishan (WZS), Tibetan (TIB), and DRC—were obtained from the Ensembl database. Phylogenetic trees were first constructed for each of the nine candidate genes across the four pig breeds (EM, WZS, TIB, and DRC). Branch models were then used to calculate the ω (Ka/Ks) ratios for the candidate genes within the EM pig phylogeny. These ratios were interpreted as follows: ω > 1 for positively selected genes, ω = 1 for neutrally selected genes (indicating neutral selection), and ω < 1 for negatively selected genes. Positive selection for each core gene associated with growth and development was inferred by comparing nested models M7 and M8 within the locus model. Likelihood ratio tests (LRTs) were conducted, and the Bayes–empirical Bayes (BEB) method was applied to identify positively selected loci with posterior probabilities greater than 99%, where LRTs were significant (*p* < 0.05). The identified sites were further validated via the mixed-effects model of evolution (MEME) and the fixed-effects likelihood (FEL) model available on the DataMonkey platform (http://www.datamonkey.org/analyses, accessed on 5 May 2024) [61]. Loci satisfying the condition β+ > α and an LRT with *p* < 0.1 were identified as positively selected loci.

### 4.9. Quantitative Real-Time PCR (qRT–PCR)

Nine core genes, including *ENPP1*, *ENPP3*, *SPP1*, *CLU*, *ITGA11*, *ITGB1*, *IQGAP2*, *BMP7*, and *F2RL2*, were selected for quantitative real-time PCR (qRT–PCR) experiments involving six tissues from EM pigs: dorsal muscle, psoas major, subcutaneous shoulder fat, subcutaneous waist fat, ovary, and spleen. The primers were designed via Primer Premier (v5.0) [62] (Table S1) and synthesized by Gold Vantage Biotechnology Ltd. (https://www.genewiz.com.cn/, accessed on 29 May 2024). Real-time fluorescence qRT–PCR analysis was conducted using a LightCycler^®^ 96 system (Roche, Switzerland). The qRT–PCR system consisted of a 10 μL volume containing 5.0 μL of SYBR Green dye, 0.3 μL of each forward and reverse primer, 1.0 μL of cDNA, and 3.4 μL of sterilized water. The qRT–PCR protocol involved initial denaturation at 95 °C for 10 min, followed by 40 cycles of denaturation at 95 °C for 15 s and annealing/extension at 60 °C for 60 s. Melting curve analysis confirmed a single peak for each PCR product, indicating specificity. *GAPDH* was used as the internal reference gene, and relative mRNA expression levels were calculated via the $2^{-△△CT}$ method.

### 4.10. Analysis of Candidate Gene Haplotypes

To conduct haplotype analysis, EM pigs and HB pigs were used as reference populations. High-quality, genome-wide SNP data obtained after merging and quality control were used to extract SNP variants within a 500 kb region upstream and downstream of the *ITGB1* gene. The extracted SNPs were converted to a Haploview-compatible format via VCFtools and Plink software. Haploview (v4.2) [63] was then applied to calculate LD values between SNPs and to define LD blocks on the basis of specified threshold values.

## 5. Conclusions

Compared with other pig breeds, the Min pig population presents relatively high genetic diversity and unique genetic structure, with significant genetic differentiation between the EM and HB subpopulations. By integrating whole-genome resequencing and transcriptome data, we identified several candidate genes associated with body size variation in Min pigs, including *ENPP1*, *ENPP3*, *SPP1*, *CLU*, *ITGA11*, *ITGB1*, *IQGAP2*, *BMP7*, and *F2RL2*. These genes were enriched predominantly in pathways related to cell signaling, metabolism, lipid deposition, and skeletal development. Notably, *ITGB1*, located on chromosome 10, exhibited specific high expression in the adipose tissue of EM pigs and was under strong positive selection. A G/A polymorphism at an SNP locus within ITGB1 showed distinct allele frequency differences between EM pigs (G = 52.94%, A = 47.06%) and HB pigs (G = 0%, A = 100%).

## Figures and Tables

**Table 1:** Population genetic diversity of 14 pig breeds.

Breed	π	He	Ho	PIC	MAF	Ne	PN	$r^{2}$
HB	0.3271	0.3154	0.3185	0.2966	0.2375	306.4400	0.1319	0.5474
EM	0.2785	0.2774	0.2794	0.2749	0.2076	276.7500	0.0153	0.5804
WB	0.2284	0.2208	0.2191	0.2372	0.1646	871.4167	0.1096	0.3510
TC	0.2157	0.2089	0.2185	0.2259	0.1533	537.5833	0.1058	0.5305
SZL	0.1943	0.1852	0.2132	0.2101	0.1384	256.0000	0.1393	0.5353
BMX	0.2075	0.2010	0.2117	0.2211	0.1487	405.7876	0.1013	0.4994
RC	0.1981	0.1925	0.1978	0.2146	0.1426	561.7826	0.0894	0.4779
EHL	0.1939	0.1909	0.1905	0.2097	0.1381	446.1739	0.0359	0.5696
DS	0.1696	0.1635	0.1895	0.1919	0.1225	248.5000	0.1003	0.5393
GX	0.1516	0.1454	0.1744	0.1745	0.1083	181.0476	0.1105	0.6066
JH	0.1432	0.1377	0.1490	0.1668	0.1023	179.1429	0.1101	0.5960
LW	0.3657	0.3565	0.3515	0.3185	0.2736	322.3200	0.0945	0.5397
LR	0.3448	0.3362	0.3290	0.3088	0.2567	312.9200	0.0932	0.5607
DRC	0.2924	0.2852	0.2765	0.2784	0.2121	276.5833	0.0893	0.6063

Abbreviations: HB, Hebao; EM, Ermin; WB, wild boar; TC, Tongcheng; SZL, Shaziling; BMX, Bamaxiang; RC, Rongchang; EHL, Erhualian; DS, Dongshan; GX, Ganxi; JH, Jjinhua; LW, large white; LR, Landrace; DRC, Duroc; π, nucleotide diversity; He, expected heterozygosity; Ho, observed heterozygosity; PIC, polymorphism information content; MAF, minor allele frequency; Ne, effective population size; PN, proportion of polymorphic markers; $r^{2}$, an important metric for measuring the degree of Linkage Disequilibrium (LD).

**Table 2:** Selection pressure on nine candidate genes.

Gene	PAML	MEME (*p* < 0.1)	FEL (*p* < 0.1)	ALL Sites	ω
*ITGB1*	433, 919	17	-	3	3.49525
*CLU*	445, 446	-	-	2	1.15395
*ITGA11*	-	14	-	1	0.06436
*BMP7*	-	-	-	0	0.20388
*F2RL2*	-	-	-	0	0.66445
*IQGAP2*	2, 3, 6, 7, 9, 12, 14, 15, 19, 20, 21, 24, 25, 29	-	-	14	0.06434
*SPP1*	-	-	-	0	0.32391
*ENPP3*	-	-	-	0	0.73189
*ENPP1*	445, 446	-	-	5	0.68489

Abbreviations: PAML, phylogenetic analysis by maximum likelihood; MEME, mixed-effects model of evolution; FEL, fixed-effects likelihood; ω, the ratio of the non-synonymous mutation rate to the synonymous mutation rate.

## Data Availability

The data presented in this study are available on request from the corresponding author without any restrictions.

## Supplementary Materials

The supporting information can be downloaded at: https://www.mdpi.com/article/10.3390/ijms26041569/s1.

## Abbreviations

The following abbreviations are used in this manuscript:

**Abbreviation**	**Definition**
EM	Ermin
HB	Hebao
SNP	single-nucleotide polymorphism
MAF	minor allele frequency
LW	large white
LR	Landrace
DRC	Duroc
WB	wild boar
TC	Tongcheng
SZL	Shaziling
BMX	Bamaxiang
RC	Rongchang
EHL	Erhualian
DS	Dongshan
GX	Ganxi
JH	Jinhua
θ_π_	nucleotide diversity ratio
F_ST_	F-statistics
XP-EHH	Cross-population Extended Haplotype Homozygosity
PE	paired-end
NGS	next-generation sequencing
QC	quality control
π	nucleotide diversity
He	expected heterozygosity
Ho	observed heterozygosity
PIC	polymorphism information content
PN	proportion of polymorphic markers
LD	linkage disequilibrium
Ne	effective population size
PCA	principal component analysis
NJ	neighbor-joining
DEGs	differentially expressed genes
PPI	protein–protein interaction
GO	gene ontology
KEGG	Kyoto Encyclopedia of Genes and Genomes
WZS	Wuzhishan
TIB	Tibetan
LRTs	likelihood ratio tests
BEB	Bayes–Empirical Bayes
PAML	phylogenetic analysis by maximum likelihood
MEME	mixed-effects model of evolution
FEL	fixed-effects likelihood
ω	the ratio of the non-synonymous mutation rate to the synonymous mutation rate
qRT–PCR	quantitative real-time PCR
