# Supplementary material for: Integration of Multiomics Data Reveals Selection Characteristics of ITGB1 That Are Associated with Size Differentiation in Pigs
Source: Int J Mol Sci. 2025 Feb 13;26(4):1569. doi: 10.3390/ijms26041569 (PMC11855449; doi:10.3390/ijms26041569)
Supplement: Supplementary file 1 [file ijms-26-01569-s001.zip › Table S1.pdf]

Gene Name	Primer Sequence
F2RL2	F:GCAAAGCCAACCTTACCCATT
	R:GAGGTAGATGGCAGGTATCAGT
ITGB1	F:ATGCTATCCCAACTACACTGGC
	R:CACCAAGGCAGGTCTGACAG
ITGA11	F:CAGCTCGCTGGAGAGATACG
	R:TTACAGGACGTGTTCGCCTC
BMP7	F:TGAGTTCCGCATTTACAAGG
	R:GTGGCTGTGATGTCAAAAAC
ENPP1	F:CTGGTTTTGTCAGTATGTGTGCT
	R:CTCACCGCACCTGAATTTGTT
ENPP3	F:CCGCATCCGAGCTCATAATATA
	R:CTTTGGCAAATCAGGAGTCAAA
SPP1	F:CAGTGATTTGCTTTTGCCTCC
	R:GGCTAGGAGATTCTGCTTC
CLU	F:CTGCTGACCTGGGAGAATGG
	R:GCTCTTCATTTGATTGTTCTATTAGGG
IQGAP2	F:TGGAAGAAGGGCTTCGGAAC
	R:CAGCCACTGGACGGTGTTAT

Table S1: qRT–PCR upstream and downstream primer sequence information.
